# Immuno-PCR in the Analysis of Food Contaminants

**DOI:** 10.3390/ijms26073091

**Published:** 2025-03-27

**Authors:** Mirjana Radomirović, Nikola Gligorijević, Andreja Rajković

**Affiliations:** 1 Center of Excellence for Molecular Food Sciences and Department of Biochemistry, University of Belgrade—Faculty of Chemistry, Studentski trg 12-16, 11000 Belgrade, Serbia; 2 Center for Chemistry, University of Belgrade—Institute of Chemistry, Technology and Metallurgy, National Institute of the Republic of Serbia, Njegoševa 12, 11000 Belgrade, Serbia; nikola.gligorijevic@ihtm.bg.ac.rs; 3 Ghent University, Faculty of Bioscience Engineering, Department of Food Technology, Safety and Health, Campus Coupure, Coupure Links 653, 9000 Ghent, Belgium; 4 Ghent University Global Campus, Ghent University, Yeonsu-gu, Incheon 406-840, Republic of Korea; 5 University of Belgrade—Faculty of Agriculture, Department of Food Safety and Quality Management, Nemanjina 6, 11080 Belgrade, Serbia

**Keywords:** immuno-PCR, food contaminant, food analysis, analytical methods, sensitivity, antibody, food contamination, food safety

## Abstract

Food safety is a significant issue of global concern. Consumer safety and government regulations drive the need for the accurate analysis of food contaminants, residues and other chemical constituents of concern. Traditional methods for the detection of food contaminants often present challenges, including lengthy processing times and food matrix interference; they often require expensive equipment, skilled personnel or have limitations in sensitivity or specificity. Developing novel analytical methods that are sensitive, specific, accurate and rapid is therefore crucial for ensuring food safety and the protection of consumers. The immuno-polymerase chain reaction (IPCR) method offers a promising solution in the analysis of food contaminants by combining the specificity of conventional immunological methods with the exponential sensitivity of PCR amplification. This review evaluates the current state of IPCR methods, describes a variety of existing IPCR formats and explores their application in the analysis of food contaminants, including pathogenic bacteria and their toxins, viruses, mycotoxins, allergens, polycyclic aromatic hydrocarbons, polychlorinated biphenyls, phthalic acid esters, pesticides, antibiotics and other food contaminants. Depending on the type of analyte, either sandwich or competitive format IPCR methods are predominantly used. This review also examines limitations of current IPCR methods and explores potential advancements for future implementation in the field of food safety.

## 1. Food Contamination

Food contamination is a significant concern for consumers and poses one of the most important challenges for food producers. It occurs when an undesired substance, potentially harmful to human health, is introduced into food during various stages of food processing. Food safety encompasses practices and measures necessary for controlling and preventing food-related contaminations throughout all stages of the food production chain, often referred to as “from farm to fork”, ensuring that consumed food is safe for human health [1]. The incidence of foodborne outbreaks highlights the need for the development of sensitive, specific, fast and reliable methods for the detection of causative agents in food samples. In addition to developing reliable analytical methods, appropriate hygiene practices must also be employed in manufacturing and domestic kitchens for the safe disposal of food and prevention of the spread of food disease [2,3].

Despite the implementation of numerous established standards, food contamination remains prevalent. Approximately 600 million cases and 420,000 deaths were attributed to foodborne diseases in 2010 [4]. They were all associated with 31 hazards present in food. Certain groups of people are more vulnerable to foodborne diseases. They include the elderly, the very young, the pregnant, individuals with suppressed immune systems, those infected with HIV and persons who take certain medications [5]. In the United States, one in six individuals experienced a foodborne illness, leading to an estimated annual loss of USD 17.6 billion due to medical expenses, productivity losses and the economic impact due to fatalities [6]. Data on foodborne outbreak-associated illnesses in the United States from 1998–2008 indicate that out of a total of 9,638,301 illnesses, around 57% were attributed to viral infections, 38% to bacterial infections, while chemicals and parasites contributed with around 2.5% each. When analyzed by food category, the majority of illnesses were linked to contaminated plants (51.1%), with produce accounting for 45.9%, followed by land animals (41.7%) and aquatic animals (6.1%) [7]. Additionally, most of the zoonoses-related foodborne outbreaks reported in 2023 in the EU were due to bacterial infections, followed by bacterial toxins, viruses, “other causative agents” (including histamine and scombrotoxin, marine biotoxins, mushroom toxins and other agents) and parasites [8]. Given the significant number of foodborne outbreaks being the result of an import of contaminated food, imported food products are subject to risk categorization to assist risk managers in prioritizing interventions at the border control [9]. The Rapid Alert System for Food and Feed (RASFF) for reporting food safety issues within the European Union was established in 2002 to enable the swift exchange of information on potential hazards in food and feed identified in member states, such as undeclared allergens, above-legal limits of aflatoxins, pesticides, or heavy metals, the presence of pathogenic bacteria, etc. Analysis of the RASFF data offers valuable insights into the prevalence of specific contaminant types within the defined time period. A review of RASFF data from 1979 to 2020 reveals that the majority of hazard notifications relates to nuts, nut products and seeds (18.55% of total notifications), followed by fruits and vegetables (14.76%) and fish and fish products (10.56%). The most commonly reported hazards include mycotoxins (21.41%), pathogenic microorganisms (17.08%) and pesticide residues (11.46%) [10]. Furthermore, an analysis of RASFF notifications concerning hazards reported over a 25-year period (1997–2021) in products from northern Mediterranean countries highlights an increasing number of notifications related to ochratoxin A in fruits and vegetables, as well as pathogenic microorganisms in animal products, including *Salmonella* in chicken, *Listeria* in cheese and *Escherichia coli* in cheese and mussels [11]. These data indicate the persistent presence of food contaminants, highlighting the importance of understanding the types of contaminants and developing effective detection methods to ensure the safety of food.

### Types of Food Contamination

**Figure 1:**
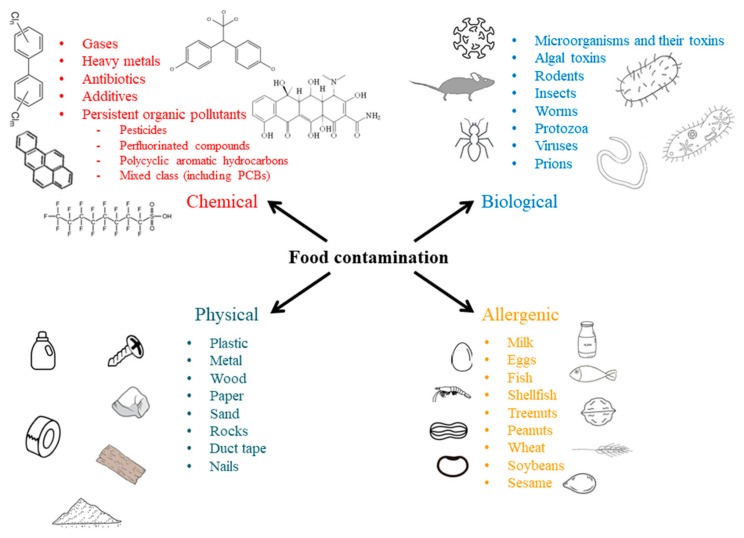
Types of food contaminants.

Food contamination can occur at various stages in the food production chain and during improper handling. Considering that food production and distribution represent a very complex chain of activities, opportunities for food contamination are many. Any substance that has been unintentionally added to food may be regarded as a contaminant. Regarding the type, food contaminants can be divided into the three main categories of biological, chemical and physical [12]. The fourth type of contamination, allergenic contamination, is sometimes considered a distinct category (Figure 1). These three categories are not always mutually exclusive since overlapping can occur, particularly between biological and chemical or biological and allergenic contaminations. In this context, allergenic contamination, mainly associated with proteins, can be classified as a subtype of the biological group of contaminants. Toxic chemicals involved in chemical food contamination, on the other hand, can be produced by different pathogens (biological contaminants).

Biological contamination occurs when food is contaminated by organisms or toxins that they produce. It remains a major cause of food-borne diseases worldwide [13]. Contaminating biological substances can be of human, rodent, insect and microorganism origin, with the latter being the most prevalent [12]. The list of diseases caused by pathogens in food is extensive with more than 200 diseases [14]. Bacterial species commonly associated with food-borne diseases include Gram-positive (*Bacillus*, *Listeria* and *Staphylococcus*) and Gram-negative (*Salmonella*, *Campylobacter*, *Escherichia coli*, *Clostridium* and *Cronobacter*) species. Fungal species associated with food contamination are strains of *Aspergillus*, *Candida*, *Penicillium*, *Fusarium* and *Mucormycetes*. Viral pathogens of concern include *norovirus*, *rotavirus* and *hepatitis* A and E. Protozoan species such as *Cryptosporidium parvum*, *Giardia lamblia* and *Toxoplasma gondii* are commonly found in livestock, while helminths, including *Echinococcus*, *Fasciola* and *Trichinella*, also contribute to biological contamination [13].

Routes for contamination of food by pathogens are multiple. Soil and water at farm sites may contain pathogens that transfer to crops and livestock. Additional risks arise during harvesting, production process, transportation, handling and processing. Processing steps, in particular, pose a higher risk of food contamination than primary production [15]. Retail sites may also contribute to food contamination due to inappropriate handling. Factors, such as shelf-life, packaging, storage conditions, the presence of rodents and temperature control, must be carefully managed to prevent contamination [16]. Excreted pathogens can spread through direct contact with humans, insects, rodents, or fluids, floors and surfaces used for food production and preparation. While some pathogens have specific transmission routes, others may be transmitted through multiple pathways, making their prevention more challenging. Such is the case for botulism, induced by *Clostridium botulinum*, listeriosis, induced by *Listeria monocytogenes* and viral gastroenteritis, caused by noroviruses [17].

Algal toxins represent a significant group of biological contaminants that can negatively impact food chains and water quality. Algal toxins are produced by specific algal species, including cyanophyta (blue-green algae or cyanobacteria), chrysophyta (class prymnesiophyceae) and pyrrhophyta [18]. Human health risks associated with algal toxins arise through multiple exposure pathways. These include the consumption of contaminated seafood, particularly shellfish, possible contamination of freshwater sources and inhalation of aerosolized toxins [19,20,21]. Rigorous monitoring and mitigation strategies are needed to reduce the risk of algal toxin exposure in food and water supplies.

Misfolded prions represent a unique form of biological contamination, as their induction occurs without the involvement of a pathogenic organism. These infectious proteins trigger the misfolding of normally folded prion proteins, initiating a self-propagating process. As misfolded proteins continue to accumulate and aggregate, they ultimately result in neural cell death [22]. Humans can contract prion disease through the consumption of infected cattle tissue [23]. Currently, no cure exists for this condition and disease progression is typically rapid following diagnosis. A major problem comes from the fact that disease is usually detected only after the first symptoms appear. Therefore, the most effective approach to combatting prion diseases is through transmission prevention. The development of reliable diagnostic tests capable of detecting prion disease prior to the onset of symptoms is of critical importance. Notable progress has been made in this regard with blood tests using real-time quaking-induced conversion and protein misfolding cyclic amplification [24].

The allergenic contamination group is unique, since, in most cases, adverse effects arise unintentionally due to the consumption of food allergens by allergic persons, although incidences of intentional exposures also occur, either due to individuals underestimating their sensitivity or knowingly accepting the risk [25]. Avoiding food containing even trace amounts of allergens is the safest way of evading possible allergic reactions, some of which can be life-threatening [26]. Within the food production chain, the unintentional cross-contact of food allergens with other food products can occur at multiple stages. Agricultural comingling, resulting from the use of shared farming fields and harvesting equipment, is one of the possible contamination sources, particularly for nut-producing farm fields. Additionally, shared processing equipment in manufacturing facilities presents another important route for possible allergen cross-contact. To mitigate these risks, food manufacturers have adopted various preventative measures. However, transportation and storage facilities are also risk points for allergen cross-contact [27].

To increase the safety of consumers, 66 countries have enacted legal requirements mandating the labeling of allergenic ingredients on their food products. The presence of major food allergens must be declared in most regions, although some countries include additional allergens, whereas others require fewer allergens on the mandatory labeling list. As a result, allergen labeling practices are not standardized and vary considerably across countries [28]. If a particular allergen is not one of the main ingredients of a pre-packed food product but may be present due to cross-contact during production, food manufactures indicate its potential presence through Precautionary Allergen Labeling (PAL) [29]. Although well-intentioned, PAL is an unregulated and voluntary practice that has led to inconsistent and often unclear labeling that frequently causes confusion and safety concerns among consumers [30]. To address these challenges, a stricter labeling framework, originating from the Australian-New Zealand Allergen Bureau, has been proposed. The Voluntary Incidental Trace Allergen Labeling (VITAL) program focuses on the quantification of allergens that were inadvertently added to food products and compares these levels with established eliciting doses or minimal amounts of allergens necessary to provoke an allergic reaction. These doses are determined based on published scientific data on the oral food challenges (OFCs) conducted for diagnostic purposes [31]. Currently, official reference doses, expressed as milligrams of proteins from allergenic sources per kilogram of food, are available for 11 allergenic sources [32].

Chemical contamination of food occurs when a substance of either natural or synthetic origin enters food products. More than 350,000 chemicals have been registered and approved for production and use; however, the identities and properties of many still remain either unknown or vaguely described [33]. A chemical is classified as a contaminant when its concentration exceeds established safety thresholds [34]. Food contamination by chemicals can occur at various stages, including food production lines, environmental exposure and food preparation [35]. Within the production line, potential sources of chemical contamination include transportation, cleaning agents, food additives and packaging materials.

The transportation process can contribute to food contamination through exhaust systems that emit gases. While packaging materials are tested for their permeability to O_2_, CO_2_ and water vapors, other gases may penetrate packaging barriers and compromise food safety [36]. Cleaning agents, while widely used and necessary for maintaining hygiene in food processing facilities, can pose contamination risks if improperly handled. These agents include peroxides, ammonium-based products and organic acids. Chlorine-based products, although cheap and effective, also react with organic matter and produce undesirable by-products such as trihalomethanes. As a result, their use has been banned in several countries [37].

Food additives are substances not primarily intended for direct consumption, but are added to processed foods to improve the safety, shelf life and sensory characteristics of food. These additives undergo rigorous safety assessment before being approved for use [38]. However, numerous studies have raised concerns regarding the potential negative effects of artificial additives and their degradation products, suggesting associations with increased risks of mental health disorders, attention deficit hyperactivity disorder, cardiovascular disease, metabolic syndrome and potential carcinogenic effects [39]. While it is suggested that some additives should be banned, others are continuously monitored and assessed for their safety.

Food packaging, while necessary for ensuring the safe storage of food, can also serve as a source of both physical and chemical food contaminations. Small particles from packaging materials, such as plastic, metal, paper or glass, can be introduced to food products and cause physical contamination. Additionally, substances like adhesives, inks and chemicals used in the production of polymers, such as bisphenol A, or coating agents, like epoxy resins used for can linings, are all possible chemical contaminants, with various negative health issues. Damaged packaging can further exacerbate the risk of contamination, especially in unsanitary environments. Routes of contamination from packaging materials include direct contact, by air, condensation and penetration of substances from the outer to the inner layer of packaging material [36,40].

In addition to contamination arising from production processes, environmental chemical pollution represents a major source of food contamination, which can occur through soil, water or anthropogenic activities. Such pollutants may be either organic or inorganic in nature. Common inorganic pollutants include ozone, nitrogen oxides, sulfur dioxide, heavy metals and fluorides. Examples of organic pollutants are persistent organic compounds, including pesticides, polychlorinated biphenyls (PCB), polycyclic aromatic hydrocarbons (PAH), dioxins and dibenzofurans, and additionally antibiotics. Naturally occurring chemical contaminants may also result from environmental processes like weathering and erosion. Anthropogenic activities, such as agriculture, industry, power generation, transportation and the use and disposal of consumer products, are also the release pathways for various chemicals [36,41].

Due to their nature and production process, some food products are more susceptible to contamination than others. This may result from various factors, such as differences in pesticide exposure, variations in plant uptake mechanisms from the environment or contaminants introduced through food packaging [42]. Additionally, individual dietary habits play a significant role in determining the level of exposure. The toxic effects of chemical contaminants depend on the nature of the contaminant, the dose received and the individual’s susceptibility and age. For example, various contaminants have been connected to an elevated cancer risk. Skin cancer has been linked to prolonged exposure to arsenic-contaminated drinking water, gastric cancer to lead contamination and liver cancer to the consumption of mercury-contaminated grain. Exposure to persistent organic pollutants during pregnancy has been linked to a higher risk of childhood obesity and increased blood pressure. Moreover, although acceptable levels for many contaminants have been established, health risk assessments for combined exposures to multiple contaminants are lacking [42]. Chronic exposure to chemicals is also an important issue that should be addressed.

Physical food contamination occurs when a foreign object accidentally enters food during the production and processing stages, transportation, packaging or retail. This type of contamination can also introduce biological and/or chemical hazards. Common contaminants include rocks, sand, metal, concrete, bones, wood, plastic, glass, fingernails, coins, glove pieces, cigarette butts, gum, nut and animal shells, etc. [43]. Micro- and nanoplastics have been the general focus lately as potential environmental pollutants that may enter the food supply. While many studies exist regarding marine ecotoxicology and microplastics in fish and shellfish, research on the occurrence of microplastics in other food types and their effect on human health are still in the early stages. The toxic effects of microplastic can occur through various mechanisms, including physical contamination by the particles themselves, chemical contamination via monomers, additives, and adsorbed chemicals, and biological contamination due to microorganisms that may colonize the particles [44].

## 2. Analytical Methods for Detection and Quantification of Food Contaminants

Consumer safety and governmental regulations drive the need for analyzing food contaminants, residues and other chemical constituents of concern. Significant efforts have been made to develop analytical tools capable of detecting contaminants in food. Most commonly used methods in food contaminants analysis are spectroscopic techniques (UV–visible, fluorescence, Raman and infrared spectroscopy), chromatographic techniques coupled with mass spectrometry (MS), different types of immunoassays (e.g., enzyme-linked immunosorbent assays or ELISA and lateral flow immunoassays), electrochemical field-effect transistors, polymerase chain reaction (PCR) and isothermal nucleic acid amplification methods and hyperspectral imaging [45,46,47]. One of the major challenges in food contaminant analysis comes from the fact that contaminants are usually present in trace amounts. Moreover, since contamination may occur at different stages, including food production, packaging, processing and consumer handling, with different types of contaminants being present in food, several analytical methods may be required for their identification. The choice of analytical approach may also vary depending on whether the possible contaminants have been anticipated or are unknown. In the first, somewhat easier case, the sample is adequately treated, and appropriate analysis is performed, depending on the nature of contaminants [35]. For instance, immunoassay techniques can be applied if contamination comes from protein allergens, but also any other analyte for which specific antibodies can be developed [48]. Conversely, if the contamination comes from microplastics, FTIR or pyrolysis-GC-MS could be the methods of choice [49]. A more complicated scenario is if the nature of contamination is unknown. In such cases, optimization of sample treatments coupled with a high-resolution mass spectrometry (HRMS) is often the best option [35].

Analytical methods for detecting biological contaminants can be broadly categorized into those that detect living organisms and those detecting their toxins. Various methods are available for the detection of living pathogens and their toxins [50]. Most commonly used and still considered a golden standard are those based on selective media to isolate and identify specific pathogens. Following culturing and isolation of pathogens, a series of biochemical and serological tests are performed to confirm microorganism identity. While highly reliable, this approach is time-consuming, often requiring several days, which presents a significant limitation. Molecular-based techniques, such as PCR and antibody-based methods, offer faster alternatives. PCR enables the amplification of specific DNA or RNA sequences to confirm the presence of pathogens with high sensitivity. Although several variations of PCR exist, the technique is highly sensitive to contamination. Additionally, careful primer selection is essential to avoid false-positive results. Immunological assays, such as ELISA, are versatile methods that can be used for the detection of both pathogens and their toxins, given that toxins are dominantly proteins [51,52]. However, several toxins, like cereulide, require more sophisticated analytical methods like LC-MS [53]. For some contaminants, such as the mycotoxin beauvericin, alternative capture molecules have been developed for specific recognition, since no antibodies are available [54]. Such synthetic molecules could possibly overcome the lack of appropriate antibodies for mycotoxin detection and quantification in ELISA-based methods. Another advanced approach is flow cytometry, which utilizes specific antibodies to label and detect pathogenic cells. For a more comprehensive discussion, several review articles cover these and other methods in more detail [51,52].

Regarding allergen detection and quantification, considering they are proteins in nature, the most frequent methods used are ELISA, PCR and mass spectrometry. While MS offers high sensitivity, it requires specialized equipment and highly trained personnel, making it less accessible for routine analysis. In contrast, ELISA and PCR are more cost-effective methods and are widely used in standard laboratory settings [55]. The three mentioned methods and their suitability for the detection of VITAL^®^ 2.0/3.0 allergen reference doses were covered in a recent review [56]. The nature of samples and allergens, matrix interferences, processing, available standards, recovery and different lots of antibodies are all possible problems for standardization and the precise determination of allergens in different foods and should be carefully considered when developing allergen detection method.

The choice of analytical method for the detection of chemical contaminants depends on their chemical nature. Persistent organic pollutants are traditionally analyzed mainly by gas chromatography, high-performance liquid chromatography and chromatography coupled with mass spectrometry. While these methods offer high sensitivity, precision, accuracy and reliability, they are also time-consuming and costly. Additionally, sample preparation before analysis can be complex and includes extraction, concentration and purification [57,58]. To address these challenges, methods that enable fast and reliable on-site detection are emerging and are based on electrochemistry, optical analysis, biotechnology, modified mass spectrometry, etc. [57]. A recently published review summarized the current state-of-the-art of rapid methods applications in monitoring food contaminants [46]. Heavy metals are mainly monitored by inductively coupled plasma mass spectrometry (ICP-MS) and atomic absorption spectrometry (AAS). Their chemical form is analyzed by atomic fluorescence spectrometry (AFS) and X-ray absorption spectroscopy (XAS). The spatial distribution of heavy metals is performed using X-ray fluorescence spectrometry (XRF) and laser ablation–inductively coupled plasma–mass spectrometry (LA-ICP-MS). Finally, the dynamics of the uptake and transport of metals is analyzed by non-invasive micro-test technology (NMT) [59]. Furthermore, chemical pollutants may also be analyzed using immunological-based techniques, provided that specific antibodies are available.

Physical food contaminants can sometimes be identified through visual inspection. Metal contamination, however, requires more advanced analytical techniques, including near-infrared (NIR) spectroscopy, thermal imaging, optical techniques, microwave based, X-ray and nuclear magnetic resonance (NMR) [60]. Microplastic analysis may require appropriate sample treatment prior to analysis. This includes the filtration of water or digestion of material. Micro-FTIR, pyrolysis-GC-MS and Raman spectroscopy are standard methods for detecting microplastics in food and water [49]. The potential of artificial intelligence tools in detecting various foreign objects, including stone and plastic fragments, has recently been shown using AI-enhanced electrical impedance tomography [61].

## 3. Immuno-PCR

As outlined in the previous section, immunological methods are applicable to a wide range of food contaminants, including living pathogens and their toxins, allergens and certain low-molecular weight (LMW) chemical pollutants. These methods rely on the specific recognition of an analyte by antibodies, with both monoclonal and polyclonal antibodies being used, depending on the assay design. Monoclonal antibodies recognizing a single epitope of an antigen are generally preferred due to their higher specificity and reproducibility, making them ideal for the specific capturing and labelling of analytes. Cross-reactivity, which can lead to false-positive results, remains a potential limitation of immunological methods, particularly when employing polyclonal antibodies, and should always be assessed. Despite this limitation, immunological methods offer the advantage of being relatively rapid and cost-effective, as they do not require sophisticated instrumentation. Various subtypes of immunological assays exist, including ELISA, lateral flow immunoassay (LFIA), immunofluorescence assay, immunomagnetic separation (IMS), immunodiffusion assays, immunochromatographic assay and gold-labeled immunosorbent assay (GLISA) [62]. Among these, ELISA is a widely used biochemical technique in the field of food safety and quality due to its simplicity and effectiveness in quantifying analytes. Compared to other instrumental techniques, ELISA requires small sample volumes and enables high-throughput parallel sample processing [48]. Traditionally, ELISA has relied on the use of polyclonal antibodies. However, over time, the field of food contaminant detection with ELISA has shifted toward the use of monoclonal antibodies due to their enhanced specificity and reproducibility [45]. The sensitivity of ELISA is limited by the quality of antibodies being used and the sensitivity of the spectrophotometer used to quantify color change. The limit of detection (LOD) of ELISA method varies among different assays but typically does not go beyond the ng/mL levels. Given that many contaminants in food are present in trace amounts, it is of crucial importance to develop methods that are not only specific but also have high sensitivity in detecting analytes of interest. Although real-time PCR-based methods are highly sensitive, their applicability for analysis of food samples is negatively influenced by the PCR inhibitors in food. It is often necessary to dilute food samples more than 1000 times to eliminate the negative matrix effect on real-time PCR [63]. By combining immunological and PCR methods, interfering substances are washed away without affecting the sensitivity of the method.

**Figure 2:**
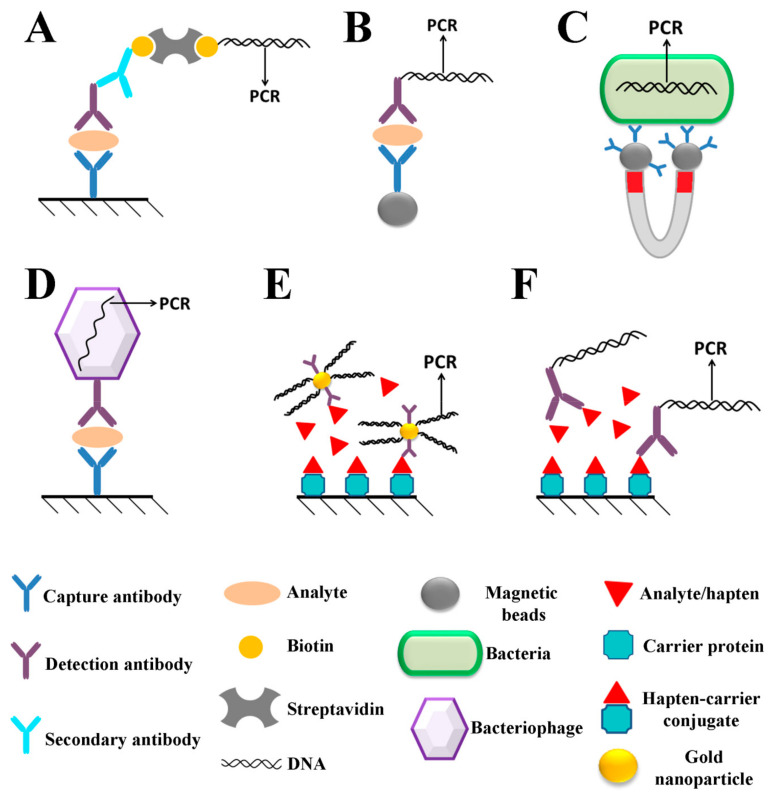
Immuno-PCR (IPCR) formats that are most commonly used in the analysis of food contaminants. (**A**) Sandwich IPCR based on streptavidin as a linker between a biotinylated secondary antibody and a biotinylated DNA molecule. (**B**) Sandwich IPCR employing magnetic beads and DNA directly conjugated to the detection antibody. (**C**) Immunocapture PCR method based on antibody-coated magnetic beads for the specific capture of target bacteria, with bacterial DNA serving as the template for PCR. (**D**) Phage-mediated sandwich IPCR in which the detection antibody is displayed on the surface of a bacteriophage. (**E**) Competitive IPCR using gold nanoparticles doubly labeled with a detection antibody and a DNA molecule for the detection of low-molecular weight (LMW) compounds. (**F**) Competitive IPCR based on DNA-conjugated detection antibodies for the detection of LMW compounds.

The immuno-PCR (IPCR) method is a relatively novel technique that was first described by Sano et al. in 1992 [64]. As its name suggests, it combines conventional immunological methods, such as ELISA, with the PCR amplification of a DNA probe. In the original method for detecting bovine serum albumin (BSA) developed by Sano and colleagues, BSA is first immobilized on the surface of a microtiter plate, after which it is incubated with a monoclonal anti-BSA antibody. Subsequently, streptavidin–protein A chimera binds to the Fc region of the monoclonal antibody via protein A, while streptavidin enables the coupling of this complex to biotinylated DNA, which is then amplified in a PCR reaction [64]. Since its discovery, immuno-PCR has evolved in different aspects and nowadays exists in various formats in which antigen capture from a complex mixture is usually achieved using antigen-specific antibodies, typically immobilized on microtiter plates or PCR tubes in a sandwich ELISA format [65,66,67] or bound to magnetic beads [68,69], although many alternative formats have also been described. After antigen binding and washing steps, the antigen is incubated with a detection antibody. Instead of the antibody–enzyme conjugate used in ELISA, the method relies on a DNA molecule usually conjugated to the detection antibody, either directly or through linkers (i.e., streptavidin, gold nanoparticles), although a DNA molecule can also be the part of the detection agent (i.e., bacteriophage DNA in phage display-mediated IPCR assays). Commonly used IPCR formats relevant for the analysis of food contaminants are schematically illustrated in Figure 2. DNA amplification is achieved through conventional end-point PCR or quantitative PCR (qPCR). Following standard PCR, DNA is visualized in agarose gel, whereas qPCR enables the real-time monitoring of amplification reaction. In non-competitive IPCR formats, the amount of DNA is directly proportional whereas in competitive formats is inversely proportional to the antigen concentration, allowing for antigen quantification in a sample of interest using a standard curve of known concentrations.

Regardless of the IPCR format, the use of antibodies ensures high specificity, while the exponential signal amplification of the PCR reaction extends the limit of detection of the immunological methods up to several orders of magnitude, therefore enabling ultrasensitive detection and quantification of the target antigen [70,71,72,73]. An additional level of specificity is secured by the use of primers specific for the DNA template, allowing for the amplification of desired sequence only, thereby reducing false-positive signals originating from possible foreign nucleic acids present in the sample. Furthermore, the use of artificial DNA sequence as a template DNA may also increase the specificity of the method. For example, Gofflot and coauthors have used a DNA construct consisting of two DNA fragments of eukaryotic and prokaryotic origin as a template for PCR [74].

Various existing IPCR formats, encompassing both traditional and innovative approaches, have already been reviewed in the literature [70,71,75]. To date, IPCR has been used for the ultrasensitive detection and quantification of various analytes, including cytokines [76,77], prion proteins [78], toxins of microbial origin [79,80,81], viral proteins [78,82,83,84], IgG antibodies to Epstein–Barr Virus [85], IgE antibodies [86], hormones such as thyroid stimulating hormone [67], biomarkers for cancer [87,88] and Alzheimer’s disease [89,90]. The developed methods have several orders of magnitude higher sensitivity in comparison to analogous ELISA formats, enabling the detection and quantification of various analytes at concentrations below the LOD of conventional ELISA methods, the factor which is of particular importance when the expected concentration of an analyte is low, or the sample amount is limited.

Although the majority of the described IPCR methods are designed for the quantification of protein antigens, the detection and quantification of any analyte against which specific antibodies can be generated is possible with immuno-PCR. The size and structural complexity of a molecule are key factors influencing immunogenicity [91]. The small size (<1000 Da) of LMW compounds and the absence of multiple epitopes in their structure required for antibody binding impose significant limitations in immunoassay format selection for their detection. However, antibody response can be generated against LMW compounds by conjugating them as haptens to carrier proteins, most commonly ovalbumin (OVA) or BSA [92]. This has prompted the development of IPCR assays for the quantification of many small molecules acting as environmental contaminants, such as PAHs [93,94,95,96] and PCBs [97,98]. Due to their small size and the absence of multiple epitopes, immunoassays designed for the detection and quantification of LMW compounds are predominantly of the competitive type in which target antigen competes with a labeled hapten to bind to surface-immobilized antibodies. To overcome the inherent limitations associated with the size of LMW compounds, in recent years, alternative non-competitive formats have also been developed [99]. Moreover, in IPCR-based approaches, hapten–protein conjugates are often used as coating agents, competing with the target analyte for binding to soluble anti-hapten antibodies [95,96,97], or as detection reagents, i.e., DNA-labeled hapten-carrier conjugates competing with the unlabeled analyte for binding to immobilized antibody molecules [100]. In the context of food safety, the antibody-based quantification of small molecules constitutes an attractive alternative to traditional techniques such as GC-MS or pyrolysis-MS that is less sensitive to matrix food interferences [101].

## 4. Application of Immuno-PCR in the Detection of Food Contaminants

Immuno-PCR has been applied for the detection of various food contaminants, including pathogenic bacteria and their toxins, viruses, mycotoxins, allergens, polycyclic aromatic hydrocarbons, polychlorinated biphenyls, phthalic acid esters, pesticides, antibiotics and other food contaminants. The literature search for IPCR-based methods was conducted using Scopus, Web of Science, PubMed and Google Scholar by using the following keywords: Immuno-PCR, immunomagnetic separation, food contamination and food contaminants. Relevant papers were selected based on their titles and abstracts and those deemed suitable were included in this study. The publication year was not considered an exclusion criterion. The principles underlying IPCR-based detection of different types of food contaminants will be described in the following sections.

### 4.1. Pathogenic Bacteria

In 2023, campylobacteriosis, with 148,181, and salmonellosis, with 77,486 confirmed human cases, were ranked as the first and second most reported zoonoses in humans in the EU. The third most reported zoonotic agent in humans was Shiga toxin-producing *Escherichia coli*, followed by *Yersinia enterocolitica* and *Listeria monocytogenes*. Although the total number of campylobacteriosis cases is almost two times higher than those of salmonellosis, the number of foodborne outbreaks of salmonellosis and cases thereof is much higher than for campylobacteriosis (1115 foodborne outbreaks and 9210 cases of salmonellosis versus 229 outbreaks and 1174 cases of campylobacteriosis). Among the most common zoonotic agents, *L. monocytogenes* infections were the most severe, with the highest fatality rates of 8.3% in 2023 for foodborne cases and 19.7% for total number of human cases, including sporadic ones [8].

The low abundance of target pathogens in food, together with the complexity of the food matrix, may influence the development of sensitive and specific methods for their detection. Among methods for detecting pathogenic bacteria in food, those combining immunological techniques with PCR stand out in terms of their insensitivity to the complexity of food matrix and to the negative influence of food matrix components on PCR amplification. Namely, traditional bacterial culture methods, although considered for a long time the gold standard in microorganism detection, are time-consuming and labor-intensive. In contrast, PCR methods are often sensitive to inhibition by food matrix components [51,52]. Although the appropriate dilution of the sample can eliminate the negative influence of food matrix components on the performance of PCR, as observed for instance in the detection of *S. enterica* in eggs and pork meat [102,103], excessive dilution may also reduce the concentration of the target antigen below the limit of detection. By using antibodies to capture antigens in immuno-PCR, the negative effect of food components on the PCR reaction is mitigated, as interfering substances are washed out.

Many described IPCR methods for the detection of different bacteria in foods comprise immunomagnetic separation (IMS) of target pathogen using magnetic beads functionalized with antibodies specific to target bacteria, followed by PCR using target-specific primers. Although such methods do not consist of a DNA molecule conjugated to the detection antibody, as in traditional IPCR methods, they could be regarded as immuno-PCR in broader sense, since they combine immunological method with the PCR amplification. In this case, however, the antigen of interest, pathogenic bacteria, already possesses genomic DNA that serves as a template in the PCR.

Immunomagnetic separation as a pretreatment technology represents an effective strategy for the selective and rapid isolation and enrichment of target pathogens from the complex food sample [104]. Several methods combining IMS with magnetic beads functionalized with anti-*Salmonella* antibodies with PCR or qPCR have been developed for specific detection of *Salmonella* in food and water samples. They are based on different genes, such as the chromosomal *invA* gene [105] or *invA* combined with plasmid *spvC* gene [106], *fliC*-d [107] or *pagC* gene [68]. Alternatively, Protein A/G can be used as an adapter for the immobilization of antibodies on the magnetic beads, such as in the case of anti-*Salmonella* antibodies in the work of Vinayaka and coauthors in which the detection of *S. enterica* in vegetable salad, eggs and pork meat was achieved with the inclusion of a Phusion hot start DNA polymerase with a high tolerance to possible PCR inhibitors. A method specificity of 100% was achieved due to the use of antibodies highly specific to “O” (somatic lipopolysaccharide antigen) and “H” (flagellar protein) antigens of *Salmonella* and *hilA* gene primers. Additionally, the achieved limit of detection was as low as ~2 CFU/mL, with the whole procedure taking less than 3 h, significantly shorter in comparison to different standard methods for detecting *Salmonella* in different food samples [103]. Moreover, target microorganisms may also be immobilized to (strept)avidin-coated magnetic beads via biotinylated pathogen-specific antibodies that can either be pre-incubated with streptavidin beads allowing for the high-affinity streptavidin–biotin interaction, followed by incubation with the sample of interest [108] or pre-incubated with the analyzed sample, followed by incubation of the antigen–pathogen complex with streptavidin beads [109]. The latter case has been reported to reduce the necessary amount of antibodies, possibly due to antibodies being more easily bound to target bacteria when in solution compared to them being immobilized on the surface of beads. Although in the referenced research IMS has not been coupled with PCR for subsequent pathogen detection, the above examples demonstrate the versatility of IMS as a strategy for the capture of bacteria. Streptavidin- or Protein A/G-functionalized beads offer more versatile applications in comparison to beads functionalized with antigen-specific antibodies, as they can be used for the detection of different type of antigens for which specific antibodies are available.

*Listeria monocytogenes* is recognized as an important foodborne pathogen. Outbreaks of listeriosis occur by the ingestion of contaminated meat, cheese, ice cream, fish and vegetables [110]. IMS in combination with real-time PCR has improved the sensitivity of *L. monocytogenes* detection in artificially contaminated milk. Capturing *L. monocytogenes* using magnetic nanoparticles covalently labelled with anti-*Listeria* antibodies, followed by real-time PCR amplifying a 113-base pair fragment of the listeriolysin O gene (*hlyA*) has enabled the detection of *L. monocytogenes* in milk samples at the level ≥10^2^ CFU/0.5 mL [111].

Multifunctional magnetic nanoparticles that are functionalized with more than one antibody type can serve for the simultaneous analysis of multiple targets, reducing in the first place the time necessary to perform these analyses separately [104]. Simultaneous detection of *Listeria monocytogenes* and *Salmonella* spp. in food samples such as milk, cheese, ice cream and beef was achieved using the combination of IMS and multiplex PCR reaction following the pre-enrichment of the samples using universal pre-enrichment broth. False negative results that could result from more than 2 log differences in the cell count of target organisms have been circumvented using equal quantities of anti-*Listeria* and anti-*Salmonella* immunomagnetic beads [112].

*Campylobacter jejuni* is the bacterial species most commonly associated with *Campylobacter* enteritis, a significant cause of morbidity worldwide. In humans, infection occurs primarily through the ingestion of contaminated food [113]. A quantitative immunocapture PCR assay for the detection of *C. jejuni* was developed based on adding polyclonal IgG capture anti-*Campylobacter* antibodies to samples containing the bacteria, followed by the addition of magnetic beads coated with secondary anti-IgG antibodies. After magnetic separation, bacterial cells are lysed and released and DNA is amplified using biotinylated primers and fluoresceinated dUTP. Obtained PCR products are detected via an avidin capture assay. In this process, separate microtiter plates are coated by avidin, allowing the biotinylated DNA to bind. Detection is achieved using anti-fluorescein antibodies labeled with alkaline phosphatase. The developed test was able to detect one bacterial cell per mL of sample and performed equivalently in both pasteurized milk and chicken skin matrices [114].

*Streptococcus pyogenes* (group A *Streptococcus*, GAS) is a human-adapted pathogen capable of causing a wide range of diseases. Diseases caused by GAS infections can be relatively mild, such as strep throat and impetigo, and also very severe and life-threatening, including necrotizing fasciitis and streptococcal toxic shock syndrome. Due to its significant global morbidity and mortality, GAS represents a major public health concern [115]. An IPCR method for the detection of GAS was developed and is based on the direct coating of sonicated bacteria onto the walls of microtiter plates. After blocking of plates, biotinylated detection antibodies were added, followed by the addition of streptavidin and biotinylated DNA. After washing, DNA was detached by incubation of plates at 96 °C for 5 min and subsequently transferred to PCR tubes for amplification. PCR products were analyzed by agarose gel electrophoresis. The method demonstrated a limit of detection of 10^−2^ cells/mL. The presence of serum and sonicated meat samples in the coating solution did not inhibit detection; however, sonicated soil supernatants interfered with amplification and GAS detection [116].

In complex, heterogeneous food matrices, the presence of low-level food contaminants can be overshadowed by other food components. The complexity of food matrix may significantly influence capturing efficiency of target antigens. For instance, the higher capturing efficiency of *Listeria monocytogenes* was noted by immunomagnetic separation from milk samples in comparison to ground beef samples, regardless of the concentration of bacterial suspension (91.2% from milk versus 25.1% from beef when 10^3^ CFU/mL was captured) and when using streptavidin-coated beads to which biotinylated anti-*Listeria* antibodies were bound and then the samples were added [108]. The food matrix effect is, however, dependent on IMS strategy and the concentration of bacterial suspension. Namely, if pre-incubating biotinylated *Listeria*-specific antibodies with the food sample, no such difference in capturing efficiencies between food matrices of different complexities was observed up to an *L. monocytogenes* concentration of 10^5^ CFU/mL. Only when the concentration of *L. monocytogenes* was 10^6^ CFU/mL, a slightly reduced capturing efficiency from lettuce samples was observed in comparison to PBS suspension [109]. Highly concentrated bacterial suspensions with over 10^7^ cells/mL of competitive microorganism have been shown to result in cross-reactivity when detecting *S. enteridis* [106]. The capturing efficiency of antibodies directly influences the sensitivity of the IPCR method. By reviewing commercially available rapid methods for the detection of foodborne pathogens, Valderrama et al. showed that their detection sensitivity is still limited by the pre-enrichment step [117]. Sample preparation strategies are therefore of critical importance for the reliable detection of food contaminants. However, even when capturing efficiencies are similar between different matrices, there may be differences in PCR efficiencies resulting in overall lower sensitivity of the method in certain matrices. For instance, a significantly lower IPCR efficiency was observed for the detection of *Salmonella* in egg whites compared to other food matrices such as vegetable salad, egg yolk and minced pork meat samples, even though capturing efficiencies were almost the same [103]. This effect can most likely been attributed to the non-specific binding of food components to target-specific antibodies.

Moreover, if performing IPCR in the presence of magnetic beads, it is necessary to consider the potential effect of beads on the PCR reaction. In a study by Vinayaka et al., PCR performance was not affected by the presence of magnetic beads, even at a concentration of 4.8 × 10^7^ beads per 10 µL of the PCR reaction. However, a slight interference of magnetic beads on the intensity of SYBR green fluorescence was observed, resulting in a delay in the Ct value of approximately 1.5 cycles in the presence of beads. To account for this effect and ensure the accurate quantification of *Salmonella* in the food samples, the authors generated a standard curve in the presence of antibody-coated beads [103].

### 4.2. Bacterial Toxins

In the European Union in 2023, a total of 893 foodborne outbreaks were attributed to bacterial toxins, representing nearly 16% of all reported zoonoses’ outbreaks. *Bacillus cereus* toxins were responsible for most bacterial toxin-related outbreaks, followed by *Staphylococcus aureus* and *Clostridium perfringens* toxins. Among all identified causative agents of foodborne outbreaks, *Bacillus cereus* toxins ranked second, following *Salmonella*. Additionally, among bacterial toxin-related outbreaks, *Staphylococcus aureus* toxins accounted for the highest number of hospitalizations, while *Clostridium perfringens* toxins were associated with the highest number of cases and fatalities [8].

One of the most important bacteria responsible for foodborne poisoning is *Staphylococcus aureus*. This bacterium has the ability to produce various extracellular protein toxins. When contaminated food is not adequately stored, the organism grows and produces toxins. Notably, *S. aureus* is able to produce more than 20 different types of staphylococcal enterotoxins (SE), with SEA and SEB being the best characterized [118].

In the initial attempt to develop IPCR for the detection and quantification of SE toxins, Rajkovic et al. established an SEB detection test based on a sandwich format consisting of polyclonal antibodies as both the capture and detection antibodies. The polyclonal antibody serving as the detection antibody was biotinylated, and following the addition of streptavidin, a biotinylated reporter double-stranded DNA was added. This DNA was subsequently amplified in real-time PCR. The method was able to detect SEB at concentrations around 10 pg/mL, with a quantification range spanning from 10 to 30,000 pg/mL [79]. A similar principle employing IgY antibodies as the capture antibodies was applied for the detection of SEB. These antibodies interact less efficiently with protein A, which is produced by almost all *S. aureus* strains, possibly leading to false-positive results in immuno-based assays. The limit of detection for this method was as low as 100 fg/mL [81]. Building on this, Fischer et al. described an IPCR method for the detection of both SEA and SEB toxins. In this method, microtiter plates were coated with polyclonal anti-SEA or anti-SEB capture antibodies. After the addition of *S. aureus* supernatants and binding of its toxins to capture antibodies, SE-specific polyclonal antibodies were added, followed by secondary detection antibodies covalently labeled with reporter DNA containing EcoRI restriction site. After cleavage of DNA by the restriction enzyme and transfer to PCR tubes, real-time PCR was performed. Using this approach, LODs were 100 pg/mL for SEA and 10 pg/mL for SEB [119]. Additionally, Panneerseelan and Muriana developed a highly sensitive IPCR method for SEA and SEB detection. The method utilizes magnetic beads coated with primary polyclonal antibodies, while the detection antibody was conjugated to a 563-bp-long amino-modified reporter DNA. The coated magnetic beads were first incubated with the sample, followed by washing and blocking using gelatin. Detection antibodies labeled with reporter DNA were then introduced. Finally, real-time PCR was performed using primers that amplify 159 bp-long DNA segment. The method achieved an exceptional limit of detection of 7.5 fg/mL for both toxins, highlighting its superior sensitivity [69]. The increased sensitivity, compared to the previously described methods, may be attributed to the fewer procedural steps and the use of magnetic beads which have higher surface area compared to microtiter plates.

Various phage display-mediated IPCR formats have been described for the detection of food contaminants, including SEA. In these approaches, phage particles are employed to display antibody fragments, including single-chain variable fragments (scFv), variable domain of heavy-chain antibodies (VHHs, also known as nanobodies) and antigen-binding fragments (Fab). These fragments replace conventional detection antibodies, while bacteriophage DNA replaces chemically bound DNA molecules [92,120]. For SEA detection, a phage display-mediated immuno-PCR method was developed with the limit of detection reported to be 100 pg/mL. Here, a monoclonal anti-SEA antibody was used as a capture antibody. After the addition of a sample containing SEA toxin, bacteriophages that expressed on their surface scFv mini antibodies against SEA were added. DNA within the phages themselves served as a template for the real-time PCR reaction. Using specific primers, the 879-bp sequence was amplified [121].

Quantitative and highly sensitive real-time IPCR was developed for the detection of enterotoxin H from *S. aureus* (SEH) [122]. In a sandwich format, the developed method had a limit of detection of 4.5 pg/mL which was almost 300 times more sensitive than the ELISA of similar format, which had a limit of detection of 1.2 ng/mL. The developed method consisted of monoclonal anti-SEH antibody as the capture and streptavidin-conjugated variant as the detection antibody. Biotinylated DNA probe containing *Bam*HI restriction site in its sequence was allowed to bind to the detection antibody, and after addition of *Bam*HI, the released DNA was amplified in a real-time PCR.

There are many commercially available ELISA kits for the detection of SE toxins, among which, some have a sensitivity as high as some of the developed IPCR methods. For example, a very sensitive ELISA kit from MyBioSource has a detection limit of 20 pg/mL [123]. The manufacturer does not specify for which SE this ELISA kit is made. Nevertheless, most of the described IPCR methods are more sensitive that this ELISA kit.

*Clostridium perfringens* is an anaerobic bacteria present in the gastrointestinal tract of humans and animals. It is also a zoonotic pathogen that can cause several diseases. Enterotoxin (CPE) causes enteritis and diarrhea while alpha toxin (CPA) causes serious life-threatening conditions like mucosal necrosis, hemorrhage enteritis, jejunal hemorrhage and enterotoxemia. A sandwich duplex immuno-capture PCR was developed for the simultaneous and differential detection of CPA and CPE from complex matrices [124]. The principle of this method is a sandwich format in which anti-r-Cpae antibody, specific for both CPA and CPE, is used as a capture antibody. After sample addition, two detection complexes, consisting of biotinylated anti-CPA (complex 1) and biotinylated anti-CPE (complex 2) antibodies and biotinylated DNA bridged by streptavidin are added. The formed sandwich is eluted from the microtiter plate by hot water and DNA is amplified and analyzed by agarose gel electrophoresis and densitometry software. In the PBS buffer, the LOD was 1 pg/mL, which was also the case for spiked fecal, muscle and serum samples. The method was somewhat less sensitive for intestinal tissue samples for which the LOD was 10 pg/mL.

*Escherichia coli* strains that produce Shiga toxins (Stx) which are a significant cause of foodborne diseases. The *E. coli* serotype O157:H7 is the most commonly associated with human disease outbreaks [125]. Shiga toxins induce diarrhea, hemorrhagic colitis and even life-threatening hemolytic–uremic syndrome [126]. These toxins are proteins composed of one A subunit and five B subunits with the ability to diminish host cell protein synthesis [127].

An IPCR method for the detection of Stx2 was developed by Zhang et al. with the limit of detection of 10 pg/mL. The method involves coating strips with Stx2 protein standard, followed by a blocking step. A biotin-conjugated detection antibody is then added, followed by the addition of streptavidin-biotinylated DNA complex containing a *Bam*HI restriction site. After detaching of DNA by the restriction enzyme, samples are transferred to new tubes for real-time PCR analysis. The method demonstrated similar detection limit in spiked urine samples; however, it could not be used for the detection of Stx2 in filtrated spiked stool samples, likely due to interference and the blocking effect of other stool components [128].

A highly sensitive and specific sandwich IPCR method for Stx2 detection was later reported by He et al. In this method, a monoclonal anti-Stx2 antibody targeting the B subunit served as the capture antibody, while a monoclonal anti-Stx2 antibody recognizing the A subunit functioned as the detection antibody. Streptavidin is covalently linked to the detection antibody, allowing for the binding of a biotinylated DNA molecule containing a *Bam*HI restriction site. Following DNA cleavage by restriction enzyme, real-time PCR was performed. The presented method was 100 times more sensitive than the previously described, direct IPCR method, with a limit of detection of 0.1 pg/mL in phosphate buffer, while for the broths from three different water samples, fecal, swine colon and soil samples detection limits ranged from 1 to 100 pg/mL [129].

*Clostridium botulinum* neurotoxins A and B (BoNT/A and BoNT/B) were detected in milk with the sensitivity higher than the one obtained in analogous ELISA by employing the sandwich method based on polyclonal anti-BoNT/A or anti-BoNT/B antibodies as capture antibodies and biotinylated monoclonal anti-BoNT/A or anti-BoNT/B antibodies as detection antibodies, with the toxoid form of the neurotoxins serving as the antigen. Biotinylated antibodies were linked to biotinylated DNA via streptavidin. The improved limit of detection of 90 pg/mL for BoNT/A and 750 pg/mL for BoNT/B in semi-fat milk was obtained in IPCR, compared to 30 ng/mL for both BoNTs obtained in ELISA. Further enhancement of IPCR sensitivity was obtained when the toxin form was used instead of the toxoid forms, yielding an LOD of 3.75 pg/mL for BoNT/A in semi-fat milk [80]. A similar set-up using a monoclonal antibody as the capture antibody and polyclonal antibody as the detection antibody demonstrated approximately four times higher sensitivity for BoNT/A detection in PBS [130], highlighting the importance of choosing the appropriate combination of capture and detection antibodies when developing IPCR assay.

### 4.3. Mycotoxins

Mycotoxins are toxic, small molecular secondary metabolites produced by various fungal species. Major groups of mycotoxins are aflatoxin, ochratoxin, citrinin, ergot, patulin and fumonisins [131]. Immunological detection of many LMW compounds is based on the use of a conjugate of haptens and carrier proteins [132]. As with many LMW compounds, IPCR detection of mycotoxins is also based on competitive formats.

Aflatoxin B_1_ (AFB_1_) is a very dangerous mycotoxin whose toxic side effects may arise even at concentrations lower than 1 pg/g. Additionally, this molecule has a very high heat and degradation resistance and has been categorized as a carcinogenic grade I by the International Agency for Research on Cancer (IARC). A very sensitive magnetic immuno-PCR method has been developed for this mycotoxin with a limit of detection of 10 fg/mL. The method consists of direct competitive immunoreaction between antigen (BSA-AFB_1_)-labeled magnetic nanoparticles and standard or samples containing AFB_1_ for binding to gold nanoparticles containing both anti-AFB_1_ antibodies and DNA. In the last step, barcode DNA is released from capture DNA and amplified using real-time PCR [133].

A competitive VHH phage display-mediated immuno-PCR assay was specifically developed for the detection of ochratoxin A in cereals. The method consisted of ochratoxin A–OVA conjugate immobilized on the wells of a microtiter plate to which a phage-displayed VHH fragment specific for ochratoxin A was bound. In the presence of ochratoxin A in the sample, the binding of the VHH phage was inhibited. Subsequently, the bound phages were subjected to thermal lysis and their DNA was quantified in real-time PCR. The developed method had an LOD as low as 3.7 fg/mL and successfully quantified ochratoxin A in cereal samples at concentrations below the LOD of a commercially available ELISA kit [134].

The chemical structure of many LMW compounds often does not allow chemical coupling to proteins or their original structure needs to be altered to be successfully conjugated to the protein carrier. For that reason and to avoid the health risks associated with the use of dangerous hapten–protein conjugates of toxins, alternative IPCR formats have been developed that make use of anti-idiotypic antibodies serving as non-toxic mycotoxin substitutes. Specifically, variable domains of heavy-chain antibodies (VHHs) are employed to generate anti-idiotypic antibodies that effectively mimic antibody binding sites of the antigen and compete with the analyte in the sample for interaction with the primary antibody [92]. One such anti-idiotypic VHH phage display-mediated immuno-PCR assay was developed for the detection of ochratoxin A in grains and food products, achieving the LOD of 4.17 pg/mL. The developed assay utilized a phage-displayed anti-idiotypic nanobody against anti-ochratoxin A monoclonal antibodies, which competed with ochratoxin A in the sample for binding to an immobilized anti-ochratoxin A monoclonal antibody [135]. Although this method was three orders of magnitude less sensitive than a previously described phage display-based assay for ochratoxin A [134], it offers the advantage of not requiring an ochratoxin A–carrier protein conjugate and is thus considered to be a more environmentally friendly alternative. Similar anti-idiotypic VHH phage display-mediated immuno-PCR assays have been developed for the ultrasensitive detection of zearalenone in cereals, with an LOD of 6.5 pg/mL [136], as well as for the detection of citrinin, a secondary metabolic by-product of *Monascus* fermentation, resulting in an LOD of 80 pg/mL [137].

For some contaminants, such as mycotoxin beauvericin, alternative capture molecules have been developed for their specific recognition [54]. Such synthetic molecules could possibly overcome the lack of appropriate antibodies for mycotoxin detection and quantification and enable the future development of alternative IPCR formats in which LMW could be in the sandwich between such synthetic receptors and the corresponding detection antibody.

### 4.4. Viruses

Reports on the application of IPCR in detection of viruses have mainly been related to early-phase detection of viral proteins in clinical samples [78,82,83,84]. However, the same methodological principles can be applied to the detection of any virus, including those associated with foodborne diseases. In that context, IPCR has so far been applied for sensitive detection of hepatitis A virus [138], norovirus [63] and rotavirus [139], all known to be implicated in food contamination. In the European Union in 2023, viruses accounted for nearly 7% of all reported foodborne outbreaks. Among these, norovirus and other caliciviruses were the predominant causes, representing 94% of virus-related outbreaks, followed by hepatitis A virus at approximately 3%. Hepatitis E virus, adenovirus, flavivirus and rotavirus comprised a minor fraction of the total virus-related outbreaks [8]. Immuno-PCR methods for detecting viral contaminants are either based on detecting viral antigens using antigen-specific antibodies conjugated with DNA (directly or through avidin) or on the specific capture of viral particles, followed by the amplification of viral genetic material in reverse-transcriptase PCR (RT-PCR). For RNA viruses, protein-based detection is advantageous due to the higher stability of proteins compared to RNA.

Norovirus is the leading global cause of viral gastroenteritis across all age groups and is responsible for around 200,000 deaths annually, predominantly in underdeveloped countries. Almost half of norovirus reported cases are related to acute care and long-term care facilities [140]. Even in developed countries, norovirus infections are prevalent, with 58% of all foodborne illnesses in the United States attributed to noroviruses [141]. Norovirus infections are often linked to the consumption of contaminated shellfish, although raw vegetables and fruits may also be contaminated through irrigation with contaminated water [142]. The sandwich IPCR method for detecting norovirus capsid proteins in fecal and food samples, such as oysters, various salads and fresh strawberries, was developed, consisting of polyclonal antisera against recombinant Norwalk viral-like particles (rNVLPs) used both as capture and detection antibodies. By bridging biotinylated secondary antibodies and biotinylated DNA through avidin and subsequent amplification of DNA, more than 1000-fold higher sensitivity than in standard ELISA and approximately 10 times higher than RT-PCR was obtained. One hundred rNVLPs, corresponding to 10 fg of purified rNVLP, and a sample containing 660 rNVLPs particle units (corresponding to 66 fg of purified rNVLP) could be detected by this method [63].

Hepatitis A virus (HAV) infection causing acute viral hepatitis is a public health issue of global concern, particularly in developing countries in which sanitation practices may be inadequate. The infection is mainly transmitted through the fecal–oral route via exposure to contaminated food or water or through direct contact with an infectious person, with contaminated food being responsible for 2–7% of all HAV outbreaks worldwide [143]. Hepatitis A viruses are resistant to common food production processes used to inactivate or control pathogens, allowing them to survive in the environment and food [144]. Various food products have been associated with HAV outbreaks, including shellfish, scallops, semi-dried tomatoes, onions, frozen berries, pomegranate arils and baked items. Despite the virus’s inability to replicate outside a host, food can serve as a vehicle for HAV transmission to humans. Consequently, methods of high sensitivity are needed to detect low titers of HAV in food samples [143]. The combination of immunomagnetic capture and PCR has been used for the detection of HAV in environmental and food samples. Specific immunomagnetic separation of HAV has been achieved using either streptavidin-coated magnetic beads to which biotinylated anti-HAV antibodies were bound or by direct coupling of anti-HAV antibodies to uncoated magnetic beads. Following HAV capture, magnetic beads were heated to release viral RNA, after which RT-PCR was performed. Regardless of the type of beads, the detection of 0.04 PFU was achieved in inoculated water and sewage samples [138]. In a similar approach using streptavidin-coated beads, 0.5 PFU of HAV was detected in spiked green onion and strawberry rinses [145].

Although rotavirus infections contribute to a smaller fraction of virus-associated illnesses in the EU, they result in more than 200,000 deaths annually worldwide, primarily in low-income countries. In children under the age of 5, rotavirus infections are the main cause of infantile gastroenteritis [146]. The rotavirus VP6 antigen was detected in stool samples using an ultrasensitive IPCR method based on a monoclonal anti-rotavirus antibody as the capture antibody and an anti-rotavirus antibody–DNA conjugate as the detection reagent. A limit of detection of 100 virus particles/mL was achieved, showing approximately 1000-fold improvement in sensitivity compared to an analogous ELISA based on an HRP-conjugated detection antibody. Interestingly, despite more procedural steps, no loss in sensitivity was observed when using the PCR-ELISA detection method compared to real-time PCR. Namely, in PCR-ELISA, DNA molecules are doubly labeled during PCR with biotin and digoxigenin using biotinylated primers and digoxigenin-labeled dUTP. After binding to streptavidin-coated plates, detection of biotin-digoxigenin DNA is enabled using enzyme-labeled anti-digoxigenin antibodies. This alternative detection system allows for DNA detection even in laboratories without access to a real-time PCR cycler [139,147].

### 4.5. Allergens

Detecting traces of allergens in food is critical for individuals with food allergies. In a study evaluating frequencies, causes and severity of accidental allergic reactions in adults with diagnosed food allergy, 37% of reactions were associated with suspected allergens not mentioned as ingredient or as a warning on the product label [148]. Furthermore, data extracted from the RASFF database show 154 notification events in 2024 for undeclared allergens present in food, of which 120 were classified as serious events [149]. Analytical methods for allergen detection play a vital role in ensuring the safety of food for people suffering from food allergies. Immunological methods, particularly ELISA, have frequently been employed for food allergen detection [150], and multiple commercial kits are available [151,152,153]. However, the potential of their integration with PCR for enhanced sensitivity of allergen detection in foods has yet to be fully explored. To the best of our knowledge, only two studies have reported the application of the immuno-PCR method for allergen detection in food samples, demonstrating an ultrasensitive detection of gliadin and tropomyosin.

Immuno-PCR for the detection of crustacean tropomyosin combines sandwich ELISA with real-time PCR amplification of a 77-base pairs-long DNA marker covalently conjugated to a secondary antibody. The use of a monoclonal anti-tropomyosin antibody as the capture antibody, alongside a polyclonal anti-tropomyosin antibody as the detection antibody, ensures high specificity for crustacean tropomyosin, while preventing cross-reactivity with molluscan tropomyosin. Compared to analogous ELISA consisting of an enzyme-labeled secondary antibody, the real-time PCR amplification of DNA marker increases the limit of quantification by a factor of 20, with the potential for even greater sensitivity if antibody labelling with DNA is performed at a higher yield. The method demonstrated high precision in a broad concentration range and accurately detected tropomyosin in spiked vegetable soup at different spike levels. Additionally, it also proved successful in detecting tropomyosin in commercial food products [65].

An immuno-PCR assay has also been developed for gliadin detection, employing direct coupling of gliadin to the wells of PCR strips, followed by its detection with monoclonal anti-gliadin antibody, which was either directly conjugated with DNA or biotinylated and subsequently linked to biotinylated DNA via streptavidin. Direct DNA conjugation resulted in higher sensitivity of gliadin detection, achieving a detection limit of 0.16 ng/mL (corresponding to 16 µg gliadin/100 g food), a threshold 30 times lower than that of direct ELISA [154].

Beyond these immuno-PCR formats, an alternative hybrid immunoassay known as immuno-rolling circle amplification (immuno-RCA) has been developed for the detection of the egg allergen ovalbumin, combining the immunological detection with DNA amplification, albeit not through a PCR reaction. In the immuno-RCA assay, OVA is captured to streptavidin-coated magnetic beads using biotinylated anti-OVA antibodies. Following OVA capture, another anti-OVA antibody conjugated to a DNA primer is incubated with the mixture. Finally, a circular single-stranded plasmid DNA, serving as the template, hybridizes with the DNA primer. Amplification is achieved by rolling circle amplification, with the resulting single-stranded DNA monitored in real time through the binding of SYBR Green II [155].

### 4.6. Polycyclic Aromatic Hydrocarbons

The application of IPCR for the detection of haptens such as PAHs and PCBs is nowadays being investigated more and more. These molecules significantly impact the ecosystem health due to their potential carcinogenic and mutagenic properties [156,157]. Polycyclic aromatic hydrocarbons are a large group of organic compounds containing two or more fused aromatic rings [157]. A group of authors has developed ultrasensitive IPCR assays able to detect concentrations at the level of fg/mL of several PAHs, including phenanthrene [94], fluoranthene [95], naphthalene [93] and benzo[a]pyrene [96]. Immuno-PCR assay for the detection of phenanthrene consisted of polyclonal anti-phenanthrene antibodies, bound to the surface of PCR tubes that serve to capture biotinylated phenanthrene molecule. Avidin is then used as a linker between biotinylated phenanthrene and biotinylated reporter DNA that is amplified in the real-time PCR reaction. The level of competition between phenanthrene in the analyzed sample and biotinylated phenanthrene for binding to capture antibodies is directly dependent on the phenanthrene concentration, enabling determination of its concentration based on the obtained Ct value for series of phenanthrene standards. The method had a detection limit as low as 5 fg/mL as well as a low level of cross-reactivity with structurally similar molecules, such as anthracene, fluoranthene and naphthalene, while even lower cross-reactivity was obtained for hydroxy- and carboxy-substituted naphthalene derivates [94].

A similar, although slightly different, method has been developed for detection of fluoranthene, consisting of fluoranthene–OVA conjugate coupled to the surface of PCR tubes. Anti-fluoranthene antibodies are added to the fluoranthene–OVA-coated tubes together with the analyzed sample. If the sample contains fluoranthene, fewer antibodies are bound. The signal of the anti-fluoranthene antibody is amplified in PCR reaction through the addition of biotinylated secondary antibodies, followed by the addition of avidin acting as a linker between biotinylated antibodies and biotinylated reporter DNA. The method also had an LOD of 5 fg/mL and showed a similar cross-reactivity trend as the one for phenanthrene determination [95]. Although the applicability of both methods was tested only in water samples, optimization of sample preparation should enable their use for the determination of PAHs in food samples as well [158].

Analogous competitive IPCR assay was developed for determination of the concentration of benzo[a]pyrene, with the exception that anti-benzo[a]pyrene antibodies were directly biotinylated, instead of using biotinylated secondary antibodies. The method had an LOD of 2.85 fg/mL and showed good agreement with GC-MS in terms of determined levels of benzo[a]pyrene in vegetables and barbecue food, although slightly higher values were measured with IPCR. Some level of cross-reactivity of anti-benzo[a]pyrene antibodies was noticed for compounds structurally similar to benzo[a]pyrene, among which those that have four or five ring show higher cross-reactivity than those with two or three rings. This cross-reactivity might have contributed to higher values of benzo[a]pyrene concentrations obtained using IPCR compared to those obtained using GC-MS [96].

Similar immuno-PCR method for the detection of naphthalene was also developed. However, instead of using SYBR Green for detecting amplified DNA, as in previously described PAH IPCR methods, a molecular beacon probe was used. The method had an LOD of 1 fg/mL. Using an analogous ELISA set-up, no naphthalene was found in the tap water, while low levels (200 fg/mL) of naphthalene were detected using IPCR, highlighting the improved sensitivity of IPCR over ELISA [93]. The developed IPCR method was validated using analogous ELISA. However, validation of the method with a method different than ELISA would give more information on the reliability of the developed method, since antibody detection used in ELISA also forms the basis of IPCR.

### 4.7. Polychlorinated Biphenyls

Polychlorinated biphenyls are a class of aromatic compounds containing one to ten chlorine atoms attached to the biphenyl rings. All PCBs are known to severely impact human health to various extents and via different mechanisms, depending on their structure, by inducing the formation of oxygen species, genotoxic effects, immune suppression, an inflammatory response, endocrine and cardiovascular effects and neurological deficits. Their production and use was banned in most countries by the 1980s; however, residual PCBs are still widely present as environmental contaminants, due to their persistent, bioaccumulative nature [156,159]. Over 90% of the total exposure of the general population to PCBs comes through ingestion of contaminated food, with animal-derived fatty foods, such as meat, fish and dairy products, contributing approximately 80% to dietary exposure [156]. Although a decline in the PCB concentration in food products has been noticed in the past two decades [160], they have gained renewed attention in the food safety field, since PCBs are inadvertently produced in some chemical formulations [161]. A variety of analytical methods are available for the analysis of PCBs in food samples, including HPLC and GC coupled to MS or electron capture detection (ECD), as well as different types of sensors, such as electrochemical, immunochemical or fluorescent sensors [156]. Conjugating PCBs to protein carriers allows the production of antibodies to PCBs and the development of antibody-based methods for their detection [162]. Due to their tendency to accumulate in adipose tissues in humans, the presence of even traces of PCBs in foods is of critical importance, thus highlighting the need for the development of ultrasensitive methods for their detection, such as IPCR.

Chen and coauthors have developed a method for detecting Tri-chlorinated PCB37 based on the competition between PCB in the analyzed sample and those bound to the surface of a PCR tube in the form of a PCB37–OVA conjugate for binding to polyclonal anti-PCB37 antibodies. Antibodies to PCB37 were obtained by immunizing rabbits with a PCB-BSA conjugate. Using avidin as a linker between biotinylated anti-rabbit detection antibodies and biotinylated reporter DNA molecule, the antibody signal was amplified. The linear sensitivity range of the assay covered more than six orders of magnitude, while the limit of detection of the method reached the concentration as low as 1 fg/mL. The developed IPCR method was validated using GC-MS and somewhat lower PCB concentrations in soil samples were determined using IPCR than with using GC-MS. However, it should be noted that the developed method showed cross-reactivity with structurally similar tetrachlorobiphenyl PCB77 congener as well as with Tri-chlorinated and tetra-chlorinated Aroclors [97]. Building on the same principle, an analogous method was developed for the determination of PCB77, with the exception that anti-PCB77 antibodies were directly biotinylated, instead of using biotinylated secondary antibodies. The calculated limit of detection was similar as that obtained for PCB37 and was determined to be close to 1.5 fg/mL [98], which was five orders of magnitude lower than the one obtained in analogous ELISA [163]. Interestingly, no significant cross-reactivity was observed with none of the congeners containing lesser number of chlorine atoms than PCB77, while the highest cross-reactivity was observed with Araclor1248 in which tetra-chlorinated PCBs predominate [98]. Moreover, in contrast to method for determining PCB37, higher concentrations of PCB77 in soil samples were obtained using IPCR in comparison to GC-MS [97], indicating that fine structural differences between PCBs may influence the suitability of the chosen detection method.

### 4.8. Phthalic Acid Esters

Phthalic acid diesters (PAEs) are widely used plasticizers. Considering their adverse effects on human health, regulatory policies have been implemented in various countries to limit PAE exposure. Phthalic acid diesters are frequently found in different water sources, necessitating reliable analytical methods for their quantification. A study by Sun and Zhuang described a sensitive and reliable method for the detection and quantification of dimethyl phthalate (DMP) in water sources [164]. The method is based on a direct competitive biotin–streptavidin amplification system using real-time IPCR. Ovalbumin-DMP was synthetized as a coating antigen for PCR tubes, while BSA-DMP served as an immunogen. Following the coating of PCR tubes, DMP standards or samples were introduced alongside biotinylated anti-DMP polyclonal antibodies, followed by the addition of streptavidin and biotinylated DNA. Measured concentrations of DMP by the developed immuno-PCR method had excellent agreement with the GC-MS method, which was used for comparison. The immuno-PCR method had a limit of detection of 1.98 pg/mL, with a linearity range spanning from 10 to 100 ng/mL. In comparison, a commercially available ELISA kit by CD Creative Diagnostics (NY, USA) for the detection of DMP in cell culture fluid, body fluids, tissue homogenates, serum or plasma has a sensitivity of approximately 1 ng/mL [165]. Furthermore, the developed immuno-PCR method is approximately five times more sensitive than the indirect ELISA method developed by Zhang and co-authors, which reports a detection limit of 10 pg/mL [166].

The same authors also developed a direct competitive gold nanoparticle-based real-time IPCR for the detection of diethyl phthalate (DEP) in food samples [167]. Similar to their approach for DMP detection, BSA-DEP was used as an immunogen, while OVA-DEP served as the coating antigen. In this method, after coating of PCR tubes, DEP and gold nanoparticles containing both polyclonal anti-DEP antibodies and DNA were added, followed by real-time PCR. The limit of detection of the developed method was 1.06 pg/mL, with a linear detection range from 4 pg/mL to 40 ng/mL. For comparison, two other highly sensitive immunoassay-based methods have been reported: an ELISA-based approach with a detection limit of 7.9 pg/mL [168] and a fluorescence turn-on assay with a detection limit of 5.6 pg/mL [169].

### 4.9. Pesticides

Immuno-PCR has also been applied for the sensitive and specific detection of pesticides in food samples. Pesticides are chemicals used for the prevention and control of different organisms such as insects, rodents, bacteria, fungi, etc. While they are important in food production, they also represent serious environmental hazards with various toxic effects on humans [170]. Gold nanoparticles (AuNPs) dual-functionalized with monoclonal anti-glyphosate antibodies and double-stranded oligonucleotides were employed for the ultrasensitive detection of glyphosate, the most widely used herbicide, via an AuNP-based bio-barcode IPCR assay. In this assay, PCR tubes are coated with an OVA-glyphosate conjugate. Glyphosate in the analyzed sample competes with the OVA-glyphosate for binding to anti-glyphosate antibodies immobilized on AuNPs. The high DNA/antibody molar ratio of the preassembled AuNP probe (around 100:1) significantly surpasses the ratio typically achieved when DNA is conjugated directly to antibodies, thereby enabling further sensitivity enhancement compared to conventional IPCR approaches. The achieved LOD for glyphosate detection was 4.5 pg/g, seven orders of magnitude lower than that of analogous indirect competitive ELISA [171]. Simultaneous detection of three organophosphorus pesticides triazophos, parathion and chlorpyrifos in apple, cucumber, cabbage and pear has been achieved using the mixture of three separate dual-functionalized AuNPs, each having different antibody specificity and double-stranded DNA. Conjugates of OVA and corresponding pesticide haptens were immobilized onto the surface of magnetic beads, with pesticides in the conjugate competing with pesticides in the analyzed sample for binding to corresponding antibody. DNA was detected using digital droplet PCR, enabling further sensitivity enhancement in comparison to qPCR. Detection limits of triazophos, parathion and chlorpyrifos expressed as IC10 (concentration of pesticide needed to achieve 10% of inhibition of binding of detection antibodies to coated conjugate) were 4, 7 and 121 pg/mL, respectively [172].

### 4.10. Antibiotics

Chloramphenicol is a broad-spectrum antibiotic that has frequently been used in the past for treating infections in both humans and animals. However, its application in humans is associated with hematotoxic side effects, particularly chloramphenicol-induced aplastic anemia. Consequently, the use of chloramphenicol in food-producing animals is not authorized in EU [173] and USA [174]. However, chloramphenicol may still be used in human medicine and in non-food-producing animals, particularly in developing countries. It may also occur naturally in plants from its production by soil bacterium and in food- and feed enzyme-based supplements [173]. Detecting trace levels of chloramphenicol in animal-derived foods is crucial for protecting consumers against the health risks associated with excessive use of antibiotics and a range of methods are already available, including HPLC, LC-MS, GC, GC-MS, spectrofluorimetry and molecular spectroscopy [175,176,177]. A competitive immuno-PCR-based assay for detecting traces of chloramphenicol in milk was developed by Tao and coauthors. The principle of their method is similar to previously described competitive assays for detecting PAHs and PCBs. In this method, chloramphenicol in the sample competes with chloramphenicol conjugated to OVA and immobilized on the surface of a PCR tube for binding to anti-chloramphenicol antibodies. However, the DNA molecule is here directly covalently bound to anti-chloramphenicol antibodies which are immobilized on magnetic beads [178]. The developed IPCR method with an LOD of 0.8 pg/mL, which, considering the density of milk of around 1.03 g/mL [179], equals to around 0.78 pg/g, offers greater sensitivity in comparison to commercially available ELISA chloramphenicol kits, which are based on antibody–enzyme conjugates rather than antibody–DNA conjugates and have LODs of 20 pg/g [180], 25–250 pg/g, depending on the sample type [181] or 100 pg/g [182], to name a few. It should be emphasized that the EFSA’s Panel on Contaminants in the Food Chain concluded that the exposure to food contaminated with chloramphenicol at levels below or at current reference point for action of 0.3 µg of chloramphenicol per kg of food is not considered to be health concern for aplastic anemia or reproductive/hepatotoxic effects [173]. In that sense, the necessity of developing a method with a sensitivity greater than those of commercially available ELISA kits is debatable.

### 4.11. Other (Miscellaneous) Food Contaminants

Polycyclic musks, 7-acetyl-1,1,3,4,4,6-hexamethyl-1,2,3,4-tetrahydronaphthalene (AHTN, tonalide) being a representative, are widely used as fragrance ingredients in the cosmetic industry. Due to their release into aquatic environments, they can contaminate living organisms in water and ultimately enter the human food chain. A study by Zhang and Zhuang describes a highly sensitive method for the detection of AHTN in water samples using direct competitive IPCR. In their method, BSA-AHTN was used as an immunogen for obtaining polyclonal anti-AHTN antibodies and OVA–AHTN conjugate was used as a coating antigen. After coating the PCR tubes, both standards/samples and single-walled carbon nanotubes with covalently attached polyclonal anti-AHTN antibodies and DNA were added, followed by real-time PCR. This method achieved an exceptionally low limit of detection of 1 fg/mL, with a linear detection range from 5 to 100 fg/mL [183]. For comparison, the same research group also published two ELISA-based methods for the detection of tonalide: one based on a streptavidin–biotin interaction with a limit of detection of 46 fg/mL and another incorporating AuNPs modified with polyamidoamine, achieving a limit of detection of 16 fg/mL [184].

Tetrabromobisphenol A (TBBPA) is a commonly used additive for the preparation of flame-retardant epoxy resins. Due to its structural similarity to thyroxin, TBBPA is classified as an endocrine-disrupting chemical. Bu et al. developed a highly sensitive direct competitive real-time IPCR for the detection and quantification of TBBPA in sediment samples [185]. This method involves coating PCR tubes with an OVA–TBBPA conjugate as the coating antigen and utilizing biotin-labeled polyclonal anti-TBBPA antibodies made by immunization of rabbits with BSA-TBBPA immunogen. Following the addition of streptavidin, biotinylated DNA is added, and real-time PCR is performed to quantify TBBPA levels. The IPCR method demonstrated an exceptionally low detection limit of 2 fg/mL, with a quantification range from 10 fg/mL to 10 pg/mL. In comparison, an ELISA method employing a similar format exhibited a significantly higher detection limit of 27 pg/mL [186], highlighting the superior sensitivity of the immuno-PCR technique for TBBPA analysis.

A phage-mediated IPCR assay based on monoclonal KG9 anti-prion protein as capture antibody and recombinant phage expressing scFV as detection antibody was utilized for ultrasensitive detection of prion protein and showed improved sensitivity compared to ELISA [78].

Estradiol-17β, a naturally present hormone, is used as a growth promoter in animals to increase meat production. Consumption of meat and meat products derived from animals treated with 17β-estradiol can induce numerous toxic effects in humans [187]. Milk products constitute around 60–80% of ingested female sex steroids, while eggs contribute to nutritional hormonal intake with 10–20%, similar to meat and fish [188]. When compared to analogous ELISA, an 800-fold increase in sensitivity of 17β-estradiol detection and an expanded working range was achieved using the universal competitive IPCR method based on polyclonal antibodies. Similar to many LMW compound detection methods, developed IPCR is based on a competition between estradiol in the sample and immobilized estradiol in the form of estradiol–OVA conjugate for binding to anti-estradiol antibodies. The concentration of 17β-estradiol was accurately detected at a concentration as low as 0.7 pg/mL [189], comparable or even more sensitive than other reported methods [187].

Salbutamol, a short-acting β-2 receptor agonist, is extensively used in humans to treat pulmonary diseases [190] and in animal feed to improve the meat-to-fat ratio [191]. However, the accumulation of β-2 agonists in the edible tissues of treated animals has been linked to toxic effects in humans after consuming contaminated meat [192]. Specific and sensitive analytical methods are therefore needed to monitor the illegal use of salbutamol. An ultrasensitive competitive IPCR assay has been developed for salbutamol detection. Unlike all previously described IPCR assays, the DNA probe is here conjugated to the protein–analyte conjugate rather than to the antibody. In this assay, a DNA-labeled BSA–salbutamol conjugate competes with the salbutamol present in the sample for binding to anti-salbutamol antibodies immobilized on the surface of the PCR tube. Compared to analogous competitive ELISA employing HRP-labeled BSA-salbutamol conjugate, a 300-fold greater sensitivity was achieved, resulting in an LOD of 21 fg/mL in buffer and 28 fg/mL in urine samples. However, a significant level of cross-reactivity with structurally related clenbuterol was observed [100]. Given the rapid metabolism and excretion of salbutamol, total salbutamol (encompassing both unconjugated and conjugated forms) is considered a more reliable target to parent salbutamol as a marker residue for detecting salbutamol levels [193]. Reactivity of antibodies to salbutamol metabolites should, therefore, be carefully considered when developing immunological assays, to avoid underestimating salbutamol levels in contaminated food.

Ricin is a highly toxic glycoprotein found in the seeds of *Ricinus communis* (castor) that has been used for the intentional food contamination in the past. Due to its potential to be used as a biological weapon, analytical methods for its detection are continuously being developed [194]. An immuno-PCR method for ricin detection based on indirect ELISA has been developed, consisting of ricin standard adsorbed to the modified polypropylene plates that efficiently bind basic proteins. The detection of ricin is achieved using a primary anti-ricin antibody and secondary biotinylated antibody. Streptavidin is used as a linker between secondary antibody and biotinylated DNA, resulting in an LOD of 10 fg/mL in PBS and human serum [195]. However, the reliability of indirect ELISA-based assay for determining ricin levels in complex food matrices is debatable, as a high level of background signal is expected in such samples. Another ultrasensitive IPCR method based on sandwich ELISA was also developed and validated for detecting ricin in complex food matrices. In this method, a monoclonal anti-ricin antibody is used as the capture antibody and a polyclonal anti-ricin antibody covalently linked with streptavidin is used as the detection antibody. To release the bound biotinylated DNA from the immunocomplex and perform real-time PCR separately from the “immuno” part of the assay, the authors used a DNA marker containing BamHI restriction site. The lack of interference from ELISA components has additionally increased the sensitivity of the PCR reaction allowing for the detection of ricin up to 1 pg/mL in PBS, 10 pg/mL in spiked egg and milk matrices and 100 pg/mL in ground beef extract, demonstrating, depending on the matrix complexity, 10- to 1000-fold higher sensitivity than analogous ELISA. Even greater sensitivity with an LOD of 10 fg/mL for detecting ricin in PBS was achieved when using polyclonal anti-ricin antibodies as capture antibodies [66]. Furthermore, it should also be emphasized that in its biologically active form, ricin is composed of A and B chains joined by a single interchain disulfide bond; isolated A and B chains are not considered toxic. To assess its potential threat, it is essential to develop assays capable of distinguishing between biologically active and inactive forms of ricin [194]. The described IPCR methods for different types of food contaminants are summarized in Table 1.

## 5. Future Perspective and Challenges of Immuno-PCR in Food Safety Analysis

In conventional IPCR assays, DNA molecules have typically been linked to detection antibodies, either by direct conjugation to detection antibodies or through linking biotinylated detection antibodies and biotinylated DNA via (strept)avidin. However, newly developed IPCR formats employing gold nanoparticles doubly labeled with antibodies and DNA have enabled even higher DNA-to-antibody ratios, further increasing the sensitivity of the method. The need for developing methods of such high sensitivity depends on several factors, including the type of analyte and its maximal permitted level in certain food types, the amount of sample available for the analysis and potential interferences from food matrix components. Increased sensitivity of IPCR is of particular importance in cases where the sample amount is low or in complex food matrices. IPCR is a robust method able to detect analytes, even in complex matrices, such as fatty food samples (meat, eggs) or stool. Such complex matrices can negatively interfere with analytical procedures. However, ultrahigh sensitivity of IPCR allows for dilution of the matrix to the level where interferences are minimized, without compromising the sensitivity of the method. The exceptional sensitivity of IPCR could therefore enable its application beyond traditional food testing, such as wet-wipe testing of possibly infected or contaminated materials and surfaces. When the expected low levels of a contaminant do not allow such high dilution factors, to account for the matrix effect, some authors have suggested the preparation of a calibration curve in the presence of a food matrix. In that case, well-characterized reference materials are needed as an adequate matrix-matched control. Moreover, if performing IPCR using magnetic beads, which is the most common approach for the detection of pathogenic bacteria, some authors have suggested including a magnetic bead control to account for the potential negative effects of magnetic beads on performance of PCR reaction or SYBR Green fluorescence.

Most reported IPCR methods have been developed for detecting protein analytes. However, several have been adapted for the analysis of low-molecular weight compounds, since conjugating them to protein carriers allows the production of anti-LMW compound antibodies. Consequently, any method for which ELISA is available could potentially be adapted to the IPCR method, if increased sensitivity is required. For instance, although ELISA kits are available for detecting numerous algal toxins, no IPCR-based detection method has been reported to date, to the best of our knowledge. Furthermore, recent studies have investigated the application of immunological methods, including ELISA, for the detection of heavy metals such as cadmium [196] and lead [197], as well as non-metal trace elements, such as selenium [198]. While the sensitivity of ELISA for detecting of metals and non-metals is generally lower than that of ICP-MS, immunological methods have the advantage of requiring less sophisticated and costly equipment, making them more suitable for routine monitoring applications. Combining ELISA with PCR could increase sensitivity of detecting metals and non-metals in IPCR, potentially approaching it to the levels of ICP-MS sensitivity.

Moreover, IPCR could be adapted for the simultaneous detection of various food contaminants in a multiplex-IPCR by using capture antibodies with different analyte specificity and detection antibodies labeled with a set of DNA molecules having different properties, allowing their differentiation during or after PCR, similarly as in a digital IPCR assay used for the simultaneous detection of 5-methylcytosine and 5-hydroxymethylcitosine [199].

While the primary advantage of IPCR lies in its remarkable increase in sensitivity, its adaptability for routine food safety monitoring presents certain challenges. Unlike ELISA-based methods, which utilize commercially available antibody–enzyme conjugates, many of the described IPCR methods rely on conjugates of antibodies or gold nanoparticles with DNA, requiring the in-house synthesis of these conjugates. The commercial availability of antibody–DNA or gold nanoparticle–DNA conjugates would significantly reduce labor time, enhance reproducibility and lower costs associated with their synthesis. Moreover, the multistep nature of IPCR increases assay length, which is a critical factor for applicability of the method for large-scale testing, contributing to higher overall cost of the method. However, automation could help streamline the process and improve its feasibility. Another important factor possibly limiting the broader use of IPCR is the requirement for expertise in both ELISA and PCR. Nonetheless, both techniques are now routinely performed in biochemical laboratories. Selecting the appropriate method should take into account the type of analyte and the required sensitivity. When sample availability is limited, the exceptional sensitivity of IPCR makes it a preferable choice.

## 6. Conclusions

This review provides a comprehensive overview of the currently available IPCR assays for detecting a wide range of contaminants relevant to the food safety field, while also providing an overview of various available IPCR formats. By combining immunological and PCR methods, IPCR provides both high specificity and sensitivity in determining low levels of present contaminants. Through the amplification of antibody signals in PCR, IPCR enhances the sensitivity of traditional ELISA methods by several orders of magnitude. Additionally, IPCR enables quantification in a broader dynamic range compared to ELISA. For food contaminants already detectable by ELISA, adaptation to IPCR is feasible when increased sensitivity is required. The continued advances in overcoming IPCR limitations and standardization efforts are expected to facilitate the integration of IPCR into routine food safety monitoring.

## Figures and Tables

**Table 1:** Immuno-PCR formats for different types of food contaminants.

Class	Species	Contaminant	LOD	IPCR Format [Reference]
Bacterial toxins	*S. aureus*	SEB	<10 pg/mL in buffer and various food samples	Sandwich [79]
		SEB	0.1 pg/mL in buffer and 1 pg/mL in milk	Sandwich with IgY antibodies [81]
		SEA	100 pg/mL in buffer	Sandwich [119]
		SEB	10 pg/mL in buffer	
		SEA SEB	7.5 fg/mL in broth medium and various foods for both	Sandwich with MB [69]
		SEA	100 pg/mL in milk	Sandwich with PD [121]
		SEH	4.5 pg/mL in buffer	Sandwich [122]
	*C. perfringens*	CPA CPE	1 pg/mL in buffer, serum, muscle and feces and 10 pg/mL in intestines for both	Sandwich duplex [124]
	*E. coli*	Stx2	10 pg/mL in buffer	Competitive [128]
		Stx2	0.1 pg/mL in buffer, 1–10 pg/mL in water and 1–100 pg/mL in feces, swine colon and soil	Sandwich [129]
	*C. botulinum*	BoNT/A	90 pg/mL in milk with toxoid 3.75 pg/mL in milk with toxin	Sandwich [80]
		BoNT/B	750 pg/mL in milk with toxoid	
Mycotoxins		AFB1	10 fg/mL in buffer	Competitive with AuNP and MNP [133]
		Ochratoxin A	3.7 fg/mL in buffer	Competitive with PD [134]
		Ochratoxin A	4.17 pg/mL in buffer	Competitive with anti-idiotypic VHH PD [135]
		Zearalenone	6.5 pg/mL in buffer	Competitive with anti-idiotypic VHH PD [136]
		Citrinin	80 pg/mL in buffer	Competitive with anti-idiotypic VHH PD [137]
Viruses	Norovirus	rNVLP	100 particles in buffer 660 particles in feces and food samples	Sandwich [63]
	Rotavirus	VP6 antigen	100 particles/mL in buffer	Sandwich [139]
Allergens		Tropomyosin	11.3 pg/mL in buffer	Sandwich [65]
		Gliadin	0.16 ng/mL in buffer	Competitive [154]
		Ovalbumin	1 pg/mL in buffer	Rolling circle amplification [155]
Polycyclic aromatic hydrocarbon		Phenanthrene	5 fg/mL in buffer	Competitive [94]
		Fluoranthene	5 fg/mL in buffer	Competitive [95]
		Benzo[a]pyrene	2.85 fg/mL in buffer	Competitive [96]
		Naphthalene	1 fg/mL in buffer	Competitive [93]
Polychlorinated biphenyls		PCB37	1 fg/mL in buffer	Competitive [97]
		PCB77	1.5 fg/mL in buffer	Competitive [98]
Phthalic acid esters		Dimethyl phthalate	1.98 pg/mL in buffer	Competitive [164]
		Diethyl phthalate	1.06 pg/mL in buffer	Competitive with AuNPs [167]
Pesticides		Glyphosate	4.5 pg/g in buffer	Competitive with AuNPs [171]
		TriazophosParathionChlorpyrifos	4 pg/mL in buffer (IC10) 7 pg/mL in buffer (IC10) 121 pg/mL in buffer (IC10)	Competitive with AuNPs and MNPs and ddPCR [172]
Antibiotics		Chloramphenicol	0.8 pg/mL in buffer and milk	Competitive with MBs [178]
Other contaminants		Tonalide	1 fg/mL in buffer	Competitive [183]
		Tetrabrombisphenol A	2 fg/mL in buffer	Competitive [185]
		Estradiol-17β	0.7 pg/mL in buffer	Competitive [189]
		Salbutamol	21 fg/mL in buffer 28 fg/mL in urine	Competitive [100]
		Ricin	10 fg/mL in buffer and human serum	Competitive [195]
		Ricin	0.01 pg/mL * in buffer, 1 pg/mL ^#^ in buffer, 10 pg/mL ^#^ in eggs and milk and 100 pg/mL ^#^ in ground beef	Sandwich [66]

Abbreviations from table: MBs—magnetic beads; PD—phage display; MNPs—magnetic nanoparticles; AuNPs—gold nanoparticles; ddPCR—digital droplet PCR. ‘*’ denotes sandwich IPCR format with polyclonal antibodies as both capture and detection antibodies. ‘^#^’ denotes sandwich IPCR format with monoclonal antibodies as capture and polyclonal antibodies as detection antibodies.
